# Implementing advance care planning in early dementia care: results and insights from a pilot interventional trial

**DOI:** 10.1186/s12877-021-02529-8

**Published:** 2021-10-19

**Authors:** Francesca Bosisio, Anca-Cristina Sterie, Eve Rubli Truchard, Ralf J. Jox

**Affiliations:** 1 Service of Geriatric Medicine and Geriatric Rehabilitation, Lausanne University Hospital and Lausanne University, Lausanne, Switzerland; 2 Service of Palliative and Supportive Care, Lausanne University Hospital and Lausanne University, Lausanne, Switzerland; 3 Chair of Geriatric Palliative Care, Lausanne University Hospital and Lausanne University, Hôpital Nestlé, Lausanne, Switzerland; 4 Institute of Humanities in Medicine, Lausanne University Hospital and Lausanne University, Lausanne, Switzerland

**Keywords:** Advance care planning, Dementia, Alzheimer’s disease, Goals of care, Trial, Feasibility, Acceptability

## Abstract

**Background:**

Advance care planning (ACP) is particularly appropriate for persons with early dementia (PWED) since it promotes conversations about dementia-specific illness scenarios, addresses inconsistencies between advance directives and patients’ observed behavior, emphasizes prospective and relational autonomy, and may be generally consistent with older persons’ decision-making needs. However, despite evidence of its benefits, ACP is yet to become widely used among PWED. In this paper, we present a dementia-specific tool developed in Western Switzerland, discuss results of a pilot trial designed to promote ACP among PWED and their relatives, and discuss the feasibility and acceptability of the intervention and the study protocol in prevision of a large scale trial.

**Methods:**

This one-arm pre-post pilot trial consisted of four visits, with visits 2 and 3 being the ACP intervention. Quantitative outcome measures during visit 1 and 4 assessed the aptitude of the intervention to support PWED autonomy and relatives’ knowledge of PWED’s preferences. Feasibility was explored according to how the recruitment procedure unfurled and based on the necessary revisions to the study protocol and healthcare providers’ reason for excluding a PWED from the study. Acceptability was assessed according to pre-post evaluations, difficulties regarding the intervention or trial participation, and pre-post qualitative interviews regarding participants’ reasons to participate to the study, satisfaction with the tool and difficulties perceived.

**Results:**

The ACP intervention itself was well received by PWED and their relatives that expressed satisfaction with the procedure, especially regarding the opportunity to discuss a sensitive topic with the help of a facilitator. Five main challenges in terms of feasibility were 1) to locate eligible patients, 2) to tailor recruitment procedures to recruitment locations, 3) to adapt inclusion criteria to clinical routines, 4) to engage PWED and their relatives in ACP, and 5) to design a trial that does not burden PWED. Despite these challenges, the intervention increased the number of advance directives, the concordance between PWED’s preferences and relatives’ decision on their behalf, and relatives’ perceived control over healthcare decisions.

**Conclusion:**

Misconceptions about dementia and ACP, in the patient, relatives, and healthcare providers, combined with structural and institutional challenges, have the power to impede research and implementation of ACP in dementia care. For this reason, we conclude that a large scale trial to test a dementia-specific tool of ACP is currently not feasible in Western Switzerland and should be endorsed in a systemic approach of ACP.

**Trial registration:**

This trial was registered in the database clinicaltrial.gov with the number NCT03615027.

## Introduction

The World Health Organization estimates that the global prevalence of dementia among people over the age of 60 years is 6–9% [1]. This rate is predicted to double by 2030 and triple by 2050 as result of population aging [1]. One consequence of this is that large parts of the population will have a family member with cognitive, emotional and/or communication impairments.

Taking care of people with early dementia (PWED) generates significant challenges for both family and professional caregivers. Cognitive impairments fluctuate and may be difficult to assess [2]. Decision-making capacity (DMC) may already be selectively or temporarily impaired due to exacerbations or acute complications; however, some PWED may retain DMC with respect to several treatment-related decisions [3].

Advance directives were developed in the United States in the 1960s with the aim to empower patients and improve professionals’ and family caregivers’ compliance with patient preferences in the event of loss of DMC. Practical experience and research suggest, however, that advance directives alone are often ineffective as they do not give adequate attention to the complex process of planning future care [4]. This is particularly true in caring for PWED. First, questions about validity and authenticity of the documents may arise [5]. Second, family members often feel unprepared to make decisions about end-of-life care on behalf of their relative even in the presence of advance directives [6–8]. Third, in the advanced stages of dementia, conflicts between anticipatorily expressed preferences and current behavior may occur, thus raising complex questions about the applicability of the advance directives in a given situation [9, 10].

The concept of advance care planning (ACP), understood as a structured communicational process facilitated and implemented by trained professionals, emerged in the US in the 1990s and has gained momentum over the following decades [11]. ACP is particularly promising for PWED since it can promote conversations about dementia-specific illness scenarios, addresses inconsistencies between advance directives and the patient’s observed behavior, emphasizes relational autonomy, and may be generally more adapted to decision-making styles and needs of older people [5, 12]. However, despite evidence about its benefits [13, 14], ACP is yet to become widely used among patients with dementia [13]. Even though a lot has been written about ACP in dementia care, only a few trials have actually investigated the feasibility and acceptability of ACP for people with early dementia. Challenges include, among others, choosing the right moment to initiate ACP, adapting the existing tools to the patient’s cognitive capacity, and designating who is responsible for initiating, guiding, implementing, and updating ACP [13, 14].

In Switzerland as in other countries, policy documents from public health authorities confirm the relevance and need regarding ACP for PWED [15, 16]. However, no specific ACP tool has yet been adapted for this population. For this reason, our team developed a dementia-specific ACP intervention and conducted a pilot trial to explore its ability to support PWED autonomy, increase the frequency and the quality of advance directives, and improve relative’s knowledge of PWED’s preferences [5]. In this paper, we present and discuss practical and ethical challenges we encountered during the pilot trial aiming to assess the feasibility and acceptability of our ACP intervention in PWED and discuss possible approaches to advance ACP in dementia care.

## Methods

### Study design

This is a pilot, one-arm interventional study with a pre-post assessment: Table 1 presents the structure of the trial, pre-post outcome measures, and the content of each part of the intervention.

**Table 1:** Study design and content of the visits

Aim of the visit:	Pre-intervention assessment	Intervention		Post-intervention assessment
		Part I	Part II	
*Leader:*	*Investigator*	*ACP Facilitator*		*Investigator*
Information and consent	x			
Eligibility	x			
Sociodemographics	x			x
Perceived control on healthcare decision	x			x
Perceived involvement in healthcare decisions	x			x
Hospital anxiety and depression scale	x			x
Decisional conflict scale (only patients)	x			x
Psychological Autonomy Inventory (only patients)	x			x
Zarit burden scale (only proxies)	x			x
Concordance between patient and proxy decision	x			x
Structured interview on values and preferences		x		x
Goals of care and advance directives documentation			x	

### Participants

The ADIA pilot trial aimed to include 20-30 patient-relative dyads. Screening and recruitment procedures and inclusion criteria reflected our willingness to recruit people at an early stage of dementia, namely people that had been diagnosed with dementia but retained sufficient decision-making capacity to discuss their treatment preferences in anticipation of a loss of decision-making capacity and to document advance directives.

Information on PWED’s cognitive status was not available since the investigators did not have access to their medical files. Hence, investigators depended on physician’s and nurses’ assessment. For this reason, since ACP entails several decisions, we made sure that PWED had sufficient decision-making capacity for each of decision ACP entailed. Following Appelbaum’s procedure [17] we checked that PWED understood and could rephrase with their own words: 1) the different options available, 2) the risks and benefits of each, and 3) the consequences of their choice.

PWED were encouraged to invite a family caregiver to participate in the trial. When no family caregivers participated in the first meeting, patients were encouraged to name a healthcare surrogate decision-maker and to invite him or her to the next meeting. Table 2 describe the criteria we used to screen PWED; we discuss below how inclusion and exclusion criteria played out in practice.

**Table 2:** Inclusion and exclusion criteria before and after discussing with the clinical staffs initially planned and as finally applied after recruitment difficulties

Initially planned inclusion criteria	Final iAdded inclusion or exclusion criteria
Older than 65 years	Unchanged
PWED that have been diagnosed with an early-stage dementia of Alzheimer’s disease aetiology	Are also included: ∙ PWED that have a dementia of neurodegenerative and/or vascular etiology ∙ People with a clinically probable neurocognitive disease ∙ People with mild cognitive impairments ∙ The PWED is informed about the diagnosis by their physician Are excluded: ∙ People with fronto-temporal dementia ∙ People with anosognosia
Montreal Cognitive Assessment (MoCA) > 20 or Mini Mental State Examination (MMSE) > 20 or Clinical Dementia Rating Scale (CDR < 1.5)	Are also included: ∙ PWED retaining sufficient decision-making capacity to document advance directives according to their treating physician or nurse. ∙ People which diagnosis was communicated at least 6 months before being contacted for the study
Showing interest in advance care planning or advance directives	Unchanged
Having the necessary French language skills to engage in conversations	Unchanged
PWED that have a close family caregiver willingly to participate to this pilot intervention	Are also included: ∙ PWED that don’t have a close family caregiver willingly to participate to this pilot intervention

### Recruitment and consent procedure

PWED were pre-screened by their caring nurse and physician in a tertiary hospital memory clinic, in a respite care facility, and in two nursing homes. In addition to recruiting in healthcare institutions, we advertised the study in a journal for older citizens and recruited by word-of-mouth.

Eligible PWED or their relatives were contacted by an investigator to confirm their interest and to set up the first meeting. The pre-intervention visit was usually organized at the PWED’s residence (their home or their nursing home) or at the respite facility. During the first meeting, the investigator provided all study participants with written and oral information about the study and answered questions, enabling them to make an informed decision about their participation in the study. Given the patients’ cognitive impairments, oral and written information were simplified based on the recommendations established by Inclusion Europe and the European Commission on Lifelong Learning Program [18]. Participants were informed they could withdraw at any moment.

### The intervention

Our dementia-specific ACP intervention was developed based on the ACP tool of the Zurich University Hospital, called ACP Medizinisch Begleitet^©^ [19]. This action-centered tool emphasizes shared decision-making about goals of care and is consistent with the Swiss legal framework as well as the ACP recommendations of the Swiss Federal Public Health Office [20]. Table 3 provides detailed information about the ACP Medizinisch Begleitet^©^ tool; supplementary material is available on demand.

**Table 3:** The ACP Medizinisch Begleitet^©^ tool

The ACP Medizinisch Begleitet^©^ tool was developed by Zurich University Hospital and Palliative Zurich + Schaffhouse. Its structure and content are inspired from a German tool called « Behandlung im Voraus planen » [21], the American « Respecting Choices^©^ » [15], and the Australian « Respecting Patients Choices^©^ » [16]. ACP Medizinisch Begleitet^©^ entails two distinct parts. In the first part, trained facilitators engage people in a structured discussion about life and death, quality of life, and past experiences with care. In the second part, people are encouraged by the facilitator to appoint a surrogate decision-maker and document advance directives in three distinct situations of lost decision-making capacity. The first situation is a sudden loss of decision-making capacity due to a vital emergency, for example cardiac arrest or acute respiratory distress, when rapid medical interventions could save the life of the person. The second situation is a loss of decision-making capacity for an uncertain period of time, for instance after a severe stroke when the patient is in intensive care and life-supporting treatment is still necessary. The third situation is a permanent loss of decision-making capacity, as in the case of long-standing unresponsive wakefulness syndrome (vegetative state) or advanced dementia. For each scenario, people are asked to choose a goal of care among “prolong life”, “prolong life with certain treatment limitations” and “comfort care only”. In order to support decision-making, the tool includes evidence-based decision aids about cardiopulmonary resuscitation, respiratory distress, dialysis, artificial nutrition, and place of death. A dedicated training to facilitate ACP with this tool is available in German and in French, in Zurich and in Lausanne respectively. The training prepares healthcare providers to broach ACP, explain ACP Medizinisch Begleitet^©^ structure and contents in lay terms, facilitate the interview, and document advance decision. Training with simulated patients helps trainees to anticipate the complexities of real-life ACP interviews. Between training sessions, trainees have to practice ACP; the examination consists in a teacher directly observing the trainees performing an ACP with one of their patients.	

For our dementia-specific intervention, ACP Medizinisch Begleitet^©^ decision aids were simplified to make them easier to read and understand for PWED. Moreover, we included evidence-based decision aids about dementia and its symptoms. The structured interview was extended to include discussion about dementia-specific scenarios. A form was added to the advance directives in order to specify surrogates’ leeway in unanticipated situations [22]. More importantly, ACP within this dementia-specific intervention also aimed at empowering the surrogate to speak for their relative.

For this trial, two palliative care nurses and a specialized educator with experience in dementia care underwent the ACP Medizinisch Begleitet^©^ certification training to serve as facilitators in the ADIA pilot intervention.

### Outcome measures

Feasibility was assessed based on how the recruitment procedure unfurled and based on the necessary revisions to the study protocol and healthcare providers’ reasons for excluding a PWED from the study. Acceptability was assessed according to pre-post evaluations, difficulties in using the tool or participating in the trial, and pre-post qualitative interviews regarding participants’ reasons to participate to the study, satisfaction with the tool and difficulties perceived.

Pre-post evaluations concerning the tool’s ability to support autonomy was assessed using four psychometric scales (the Hospital Anxiety and Depression Scale, the Decisional Autonomy Scale, the Decisional Conflict Scale, and the Zarit Burden Scale, see Table 4) and two visual analogue scales for perceived involvement in and control over healthcare decisions. The tool’s ability to increase advance decisions and improve relatives’ knowledge of patient preferences was tested by counting the number of surrogate decision maker appointments, the number of advance directives before and after the intervention, and the number of PWEDs’ and surrogates’ concordant decisions in two hypothetic written scenarios [23].

**Table 4:** Psychometric scales

Based on the literature review [24–26] we selected three psychometric scales to test pre-post variations in participants’ emotions and behavior. The **Decisional Conflict Scale** [27] is frequently uses in ACP trials in order to assess decisional conflict about medical decisions before and after the intervention. The scale was translated in French [28] and comprises 16 items that participants have to rate on a 5-point Likert scale (0 = strongly agree to 4 = strongly disagree). The **Decisional Autonomy Scale** [29] is a French-speaking scale developed in Québec that contains 28 questions that covers various aspects of independence in daily life. Respondents indicate how often (from “never” to “always”) they act according to a statement. Examples of statements are: “I am satisfied with the actions I take”, “I act according to my character”, “I choose activities that will help me keep my intellectual form”, “I prefer to do it myself- even all the things that are possible for me”. The **Zarit Burden Scale** [30] measures relative’s perceived burden of care. It consists of 22 items rated on a 5-point Likert scale that ranges from 0 (never) to 4 (nearly always) with the sum of scores ranging between 0 and 88, higher scores indicating greater burden. The **Hospital Anxiety and Depression Scale** (HADS, 27) was introduced in the protocol since the IRB expressed the concern that ACP might increase distress among participants. HADS is a standardized tool that exists in French and is used frequently to assess anxiety and depression among in- and out-patients. The is composed of statements relevant to either generalized anxiety or depression, the latter being largely (but not entirely) composed of reflections of the state of anhedonia. Each item had been answered on a four-point likert scale (0–3), so the possible scores ranged from 0 to 21 for anxiety and 0 to 21 for depression, 0 meaning being not very anxious or depressed and 21 meaning being very anxious or depressed.	

More detailed information about the methodology is provided in the clinical trials international database clinicaltrials.gov.

### IRB approval

The study protocol was submitted to the local IRB (Commission d’éthique de la recherche du Canton Vaud). All methods were performed in accordance with the relevant guidelines and the Declaration of Helsinki. Written informed consent was obtained by each PWED and relative included in this study.

Even though the research was classified as a low-risk non-invasive clinical trial according to the law on human research, particular scrutiny was applied. Since the IRB had the concern that ACP may cause distress to participants, it required formal consent of the patients’ primary care physicians or nurse to prescreen and contact PWED, proof of the involvement of a psychiatrist as co-investigator, as well as an emergency response plan to address psychological distress that might emerge during ACP. Based on these adaptations, the study received IRB approval 4.5 months after the first submission.

## Results

### Feasibility

**Fig. 1:**
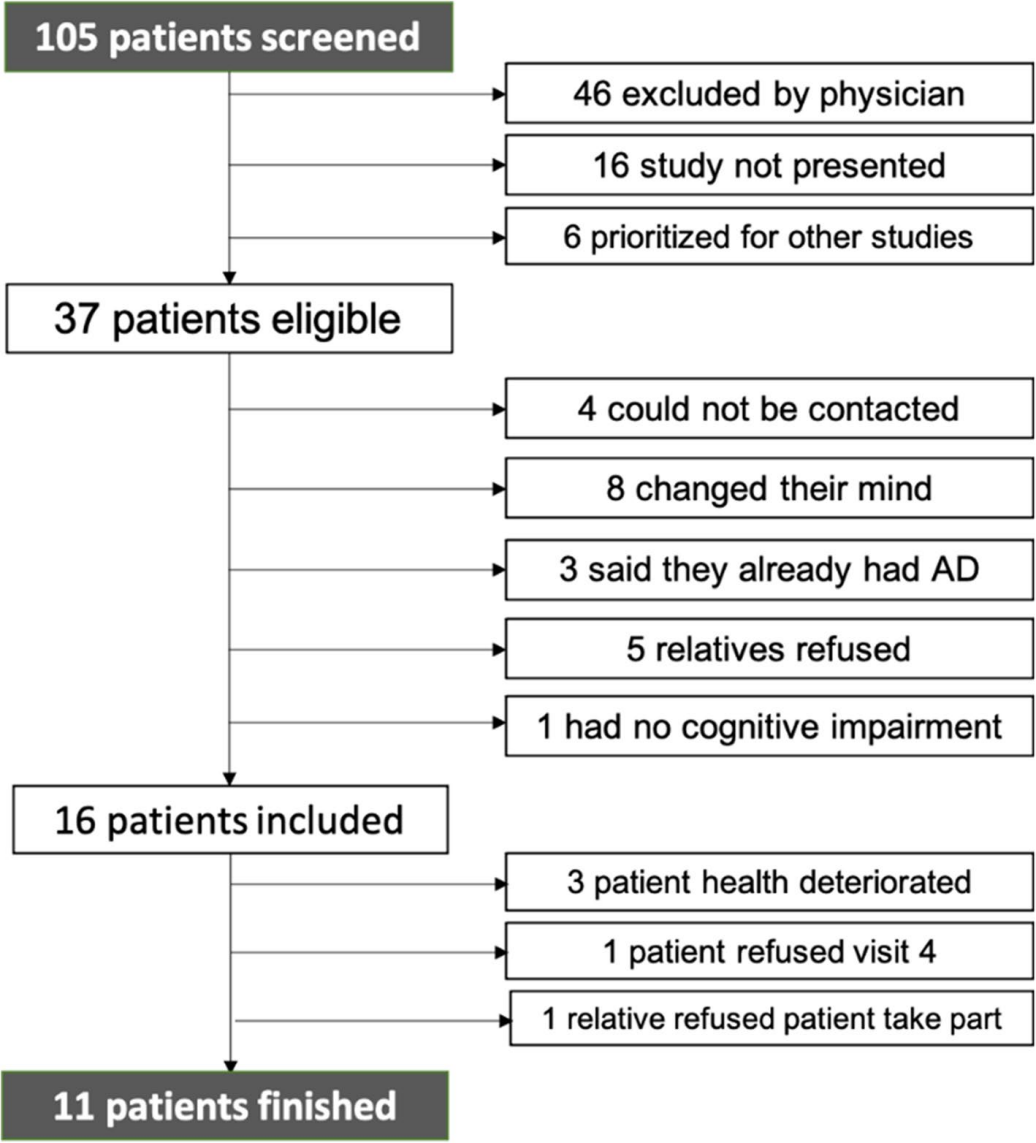
Reasons for attrition from screening to study termination. AD = advance directives

Based on the inclusion and exclusion criteria (Table 2), 105 PWED were identified as potentially eligible for the study; 11 patients and nine relatives participated to the study from August 2018 to April 2020. Fig. 1 depicts reasons for attrition. Main challenges in terms of feasibility were to locate eligible patients and secure staff’s collaboration. Three sites – a tertiary referral medical center with a geriatric unit and a memory clinic, and a regional Alzheimer’s disease advocacy group - were deemed promising to identify eligible patients for this study. However, only a low number of participants could be included. Among the 105 patients screened for participation, 46 were excluded by their physicians with variable reasons, namely, competition for various research projects, overly restrictive assessment of decision-making capacity, and ambivalence about the usefulness and ethical justification of ACP. Moreover, no one was referred by the social workers of the Alzheimer’s disease advocacy group in the first months of the study.

Hence, we decided to include two additional recruitment sites to increase the potential of eligible patients (a respite care facility and two nursing homes) which resulted in identifying 20 eligible PWED and the inclusion of eight of them. In addition, we decided to advertise our study in a journal for the elderly and to recruit word-of-mouth, which resulted in identifying four eligible people though none were included. Table 5 summarizes the number of people eligible, of those included and of those that concluded the pilot trial by locations.

**Table 5:** People Study participants included by location

Location	N eligible	N included	N completed
Memory clinic and Geriatric and rehabilitation unit	81	8	7
Alzheimer’s disease advocacy group	–	–	–
Respite day care and nursing homes	20	8	4
Word-of-mouth	2	0	0
Advertisement	2	0	0
**Total**	**105**	**16**	**11**

Among the 37 remaining eligible patients, four could not be contacted, eight changed their minds, three said they already had advance directives and didn’t feel the need for ACP. One person recruited by word of mouth was excluded since we were not able to confirm that he was diagnosed with dementia. Five relatives refused that the PWED takes part in the study. It is also noteworthy to highlight that PWED in respite care facilities were sometimes not aware of their diagnosis even though they were experiencing cognitive impairments and their professional caregivers had confirmed their eligibility for the study.

It is also worth mentioning that four proxies asked whether they could benefit from ACP without taking part in the study since they felt the procedure was too long: three of them accepted only after having been informed that the intervention was only available within the pilot trial. One PWED also refused to participate due to study length. Framing the study as a research aimed to improve care for PWED was a successful strategy to engage people in the pre-post assessment, possibly because it leveraged empathy to help advance research and other people benefit of ACP.

Among the 16 patients included, three withdrew during the course of the study because their health deteriorated, one refused to take part in visit 4 and one dropped out because his relative refused that he took part in the study. No subject dropped out due to insufficient or degrading decision-making capacity.

Table 6 presents the socio-demographic characteristics of participants. Median age of PWED and of the participating family caregiver was 81 and 62 years respectively. PWED lived alone or with a relative (*N* = 5 in both cases). Five PWED had a certificate of apprenticeship, 2 had a federal certificate and 2 a university degree. Most PWED were men whilst designated main caregivers were all women (6 were spouses and 3 were daughters). Most ACP discussions (*N* = 6) involved a PWED accompanied by a relative. In three cases, the PWED was accompanied by two relatives. In the latter case, we asked the one that self-identified as main caregiver to fill in the scales. In all cases the person designated as the main caregiver was a woman (six were spouses and three were daughters). Two PWED participated alone. Table 6 displays the socio-demographic characteristics of PWED and their main caregivers.

**Table 6:** Sociodemographic characteristics of the participating dyads

	Patients (*N* = 11)	Relative (*N* = 9)
Median age	81	62
Women	5 (45%)	9 (100%)
Degree:		
- Mandatory school	1 (9%)	1 (11%)
- Certificate of apprenticeship	5 (46%)	3 (33%)
- Federal certificate	3 (27%)	2 (22%)
- University or HES or EPFL	2 (18%)	3 (33%)
Patient lives:		
- Alone	5 (45.5%)	
- With a relative	5 (45.5%)	
- In an institution	1 (9%)	
Main caregivers are:		
- Spouses		6 (66%)
- Daughters		3 (33%)

### Acceptability

Acceptability was assessed according to pre-post evaluations, difficulties observed regarding using the tool or participating in the trial, and pre-post interviews regarding participants’ reasons to participate to the study, satisfaction with the tool and difficulties perceived.

#### Pre-post evaluations and difficulties observed regarding using the tool or participating in the trial

Table 7 presents pre-post outcomes measures. Median levels of anxiety and depression were low before and after the intervention for both PWED and their relatives, as was relative’s perceived burden (see Table 7). Important results of the intervention were to increase concordance between PWED choices and relatives’ guess (83% concordance before the intervention and 100% after), presence of advance directives (2/11 before the intervention and 10 out of 11 after it), and designation of a surrogate decision-maker (4/9 before and 9/11 after the intervention).

**Table 7:** Outcome measures (means unless said specified otherwise)

	Pre	Post
Hospital anxiety and depression scale:		
- Median anxiety (min/max):		
∘ Patient (*N* = 9)	5 (3/9)	6 (1/7)
∘ Proxy (*N* = 9)	5 (3/6)	3 (3/7)
- Median depression (min/max):		
∘ Patient (*N* = 9)	6.5 (3/11)	4.5 (2/11)
∘ Proxy (*N* = 9)	8 (3/12)	8 (4/12)
Mean Zarit Burden Score (relative only, *N* = 9)	28.28	31.83
Concordance between patient preferences and surrogate decision:		
- Scenario 1 (*N* = 6)	5 (83%)	6 (100%)
- Scenario 2 (*N* = 6)	5 (83%)	6 (100%)
Mean relative’s perception of being in control from one (no control) to 10 (full control) (*N* = 9)	5.83	8.16
PWED advance directives present, n (%) (*N* = 11)	2 (18%)	10 (90%)
Surrogate decision maker designated, n (%) (*N* = 11)	4 (36%)	9 (81%)

During the pre- and post-intervention visit, we observed that most PWED struggled with the psychometric assessment scales. The Psychological Autonomy Inventory was judged to be long and the PWED did not understand all the questions. Most PWED had problems filling in the Decisional Conflict Scale because they could remember going through the ACP process but could not recall particular steps or decisions. One spouse also reported being hurt by the Zarit burden scale since she felt caring for her partner should not be a burden at all. Several other relatives also reported that the study entailed too many questionnaires and scales.

With regard to the difficulties mentioned above, investigators decided to assist PWED with filling in questionnaires and scales. Decision was also made to prioritize the HADS, visual analogues scales on perceived control over and involvement in healthcare decisions, and the concordance between patient and their relatives’ decisions on specific healthcare scenarios. Despite this adjustment, we observed a large amount of missing data, particularly in PWED questionnaires (Table 7). Altogether these experiences suggest that scales tend to burden PWED and make them feel uncomfortable.

#### Pre-post interviews regarding participants’ reasons to participate to the study, satisfaction with the tool and difficulties perceived

Table 8 presents exemplary excerpts of the pre-post interviews. During the pre-intervention interview, participants were asked about reasons to participate in ACP. The main reasons relatives and PWED alike brought up for their study participation were difficult experiences with the end of life of a close relative and the need to “make things easy” for the relatives (excerpt 1, 2 and 3). Even though the first interview didn’t specifically investigate existing preferences about the end of life (just the existence of advance directives), five participants spontaneously mentioned their wish not to undergo futile care (excerpt 6 and 7) before this had been discussed with a facilitator. Difficult relations with estranged family members who should be surrogate decision makers was also given as a reason for participating in the study. Four dyads referred to having difficulty communicating about this topic due to the emotional charge of the discussion, either among the couple (excerpt 4) or between them and their children (excerpt 5). This was presented as a reason for participating in ACP or as a reason for not sharing (yet) the decisions with their children. Other reasons to engage in ACP included age, health status, and, for one participant, the hope that participating in this study would result in better treatment of his recently diagnosed Alzheimer’s disease.

**Table 8:** Exemplary excerpts from qualitative data

Topics	Occurrences	Exemplary excerpt
End of life or death of a close one	5	(1) “Well, there was your brother, that became suddenly sick with a brain hemorrhage … he was left 4 months without speaking, being able to move, walk, nothing … Heu, I was pained by that situation (…) and after all, he had a chemotherapy anyway …” (D1V1, relative) (2) “There was my brother’s wife … so, her son, was on artificial nutrition. It stroke me … » (D6V1, relative)
Difficulties communicating on this topic	4	(3) “You (PWD) don’t like to speak about that … seriously, when we are only the both of us. So … when there’s someone else (the ACP facilitator), it helps...” (D1V1, relative to PWD) (4) “Our children, they are a little bit avoiding this conversation. (…) Yesterday, we said to our son that we would meet you this morning … and suddenly his expression changed, he shuttered us out. We feel that in his opinion speaking about that might bring us all bad luck” (D4V1, relative)
Preference for no futile care	5	(5) “Well, our treatment preference is … no futile care … it (dying) should be quick” (D2V1, PWD) (6) « Relative: Actually, my mother, she always said that she didn’t want futile care to keep her (alive) …” “PWD: Yeah, I told you all that a long time ago.” (D16V1)

Post-intervention interviews investigated participants’ satisfaction with the dementia-specific tool. Among the elements most appreciated, participants noted the facilitators’ technique – the fact that facilitators were agreeable persons, punctual and flexible in terms of schedule, their way of explaining things (for example by giving examples from their actual experience), thus facilitating discussions that patients would have only reluctantly had with their partners. Four participants also appreciated elements related to the structure of the ACP discussion, such as the opportunity to discuss values before decisions. The mere opportunity to document decisions with the support of a professional was noted as the main element appreciated by four participants. Several patients also referred to ACP’s beneficial impact on the relationship with the relative participating in the discussion. Two participants mentioned that ACP allowed them to engage in a difficult discussion together and learn something more about one another. Three participants noted that documenting preference also resulted in a relief, either for the patient or for the proxy. Other results noted were that ACP set in motion other type of advance decisions – such as funeral arrangements – or encouraged participants’ acquaintances to document their advance directives. Among the main difficulties underlined was the complexity of the ACP part on medical treatment decisions, for example the lacking clarity of the questions, the use of percentages to indicate the likeliness of survival, and the complicated way in which options about future care were presented. Two PWED felt that some formulations were too complicated and needed their relatives to “translate” them.

## Discussion

We present and discuss practical and ethical challenges we encountered during the pilot trial and our strategy in dealing with them following three main axes: engaging PWED and their relatives in ACP, gatekeeping by professionals, and designing trials that support PWED autonomy.

### Engaging PWED and their relatives in ACP

There are several ways to explain our difficulties in engaging PWED and their relatives in ACP.

Firstly, lack of awareness about advance directives was evidenced by three people saying that they already had advance directives but were unable to recall what kind of document they filled in, when they documented them, or where they were stored. This difficulty in engaging PWED and their relatives in ACP is related to a general lack of awareness among the elderly in French-speaking Switzerland about the tools that allow people to anticipate healthcare decisions [31]. Shared decision making is not a standard in Switzerland neither, particularly for the elderly and PWED [32, 33]. Hence, people might be hesitant whether it is appropriate for them to express to their physician the wish of planning ahead for loss of DMC. The fact that most people that concluded the study were identified by their physician suggests that physician's recommendation have the power to improve engagement in ACP [24].

Secondly, caring for PWED poses a significant challenge to relatives, and daily care planning tends to take precedence over advance care planning [6]. This might explain why some relatives were concerned about the length of the study. Yet, prioritizing actual care planning might also conceal a lack of knowledge about the health trajectory of PWED [34]. Indeed, we observed that, in addition to usual barriers to ACP, PWED and their relatives tend to avoid planning ahead for various reasons, including: a strong need to stay focused on the present time to circumvent acknowledging the progressive deterioration of PWED’s mental health; the PWED and relatives’ belief that this acknowledgment would be upsetting; and PWED lack of interest for the future and the expectation that family members will take care of issues as they arise [34].

Thirdly, it is worth noting that several PWED we met in respite care were not fully aware of their diagnosis. This might suggest that the diagnosis and stages of dementia were not always explained in a timely and comprehensible manner nor were they fully understood by the patients and their relatives [35, 36]. The literature also highlights that it might be difficult for healthcare providers to assess PWED knowledge about their diagnosis and that providers sometimes hesitate to initiate the process of information and disclose sensitive information [37]. Yet, partial information or non-disclosure of the diagnosis disempower patient and their relatives [6, 38]. ACP provides PWED and their relatives with an opportunity to obtain more information about the disease, its likely course, expected problems, and therapeutic options.

### Gatekeeping by professionals

End-of-life-related research, particularly with vulnerable people, presents numerous methodological challenges [32, 33]. Whereas most of them were expected, we encountered substantial unanticipated gatekeeping by the local IRB and healthcare professionals throughout our pilot study. Two reasons might explain it: firstly, it appeared that physicians had diverse appreciation of patient decision-making capacity [39] and assume the right to choose in their patients’ best interest [35, 40]. Actually, even though it is not clear in what way the fluctuation of cognitive impairments impacts decision making capacity [41], the fact that about half of the patients screened were excluded by the physicians suggests an assessment of eligibility by adding implicit supplementary criteria [36, 42].

Secondly, physicians’ reluctance to include PWED in this pilot study can be explained by conflicting roles as health professional and researcher [35, 40]. The distinction between ACP and traditional advance directives was also unclear to many health professionals who therefore might not have seen the benefit of it from a clinical perspective.

Our screening and recruitment procedure made our study and its participants particularly vulnerable to gatekeeping. This phenomenon and its consequences are well described in palliative care research, particularly with vulnerable persons [35, 37, 40]. In addition to prevent PWED to benefit of innovative approaches, gatekeeping results in sampling biases that prevent researchers from validly assessing the interventions and generalizing results, and, in our case, discuss possible application of the tool to people at more advanced stages of dementia.

These findings suggest that the feasibility of a large scale trial of a dementia-specific tool of ACP in Western Switzerland depends on a systematic approach to ACP. Consistent efforts should be provided at a national level to raise awareness about ACP in the general population, patients, and their relatives. On a more practical level, four effective recruitment strategies were: improving professionals’ awareness about ACP and its benefits, systematic screening of patient by a researcher, thoughtful messaging to show the important of the research study, and seeking the support from clinical champions [36]. Moreover, it will be important in the future to include in the design of the overall trial procedure at least part of the staff that will be involved in the screening.

### Designing trials that support PWED autonomy

Our findings suggest that our dementia-specific tool of ACP has been perceived as acceptable by the participants’ point of view. This study confirms that it improves care planning in anticipation of a loss of DMC since it increases concordance between PWED and their relatives’, and the number of advance directives and designations of a surrogate decision-maker. PWED and proxies expressed satisfaction with the procedure, especially with the opportunity to discuss these issues with the facilitator. High-quality trials demonstrate the potential of ACP understood as a longitudinal conversation to help future surrogates prepare for in the moment decision making [14, 34, 43]. These studies support using a broader (and more fitting) range of outcomes than prior work, including surrogate preparedness.

The results also suggest, however, that trials following a pre-post design with quantitative outcome measures may not be the most appropriate to PWED and their relatives. Indeed, despite PWED had sufficient DMC to participate to ACP, questionnaires put their cognitive capacities and attention span on strain. In addition, the length of the study also posed organizational challenges to family caregivers. Such challenges have already been described in the literature but effective strategies to address them are still missing [44].

Our experience advocates for study designs that are more mindful of PWED needs. We observed that visual analogue scales proved much easier for PWED. Well accepted were also interviews and the two hypothetic scenarios by which we tested the concordance between PWED decisions and surrogate decision on their behalf. Interviews were also much appreciated. We thus recommend using outcome measures that focus on the current thoughts and feelings of PWED and on concrete experiences and decisions. This might imply adapting existing tools or creating new ones. Outcome measures should focus on investigating relatives’ preparedness to make decision on the behalf of the PWED since much of the existential burden of healthcare decision will fall on relatives' shoulders [43].

## Conclusion

This study aimed at testing the feasibility and acceptability of a dementia-specific ACP intervention and its study protocol for PWED and their relatives. Findings suggest that the tool was well received by PWED and their relatives. However, the length of the overall trial, outcomes measures, and misconceptions about dementia and ACP, combined with structural challenges in institutions have the power to impede research in this field and suggest that a larger scale trial is currently not feasible in Western Switzerland.

Our findings, particularly regarding healthcare providers’ reluctance to broach ACP to PWED and their relatives, suggest that consistent efforts should be made to increase healthcare providers awareness and training. Research may focus on implementation and carefully consider how to articulate ACP with practices and mainstream frameworks, such as goal-oriented care and shared decision-making models.

## Data Availability

The datasets used and/or analysed during the current study are available from the corresponding author on reasonable request.

## Abbreviations

ACP: Advance care planning
Covid-19: Corona Virus Disease
DMC: Decision-making capacity
PWED: People with early dementia
